# Tolerability of COVID-19 Infection and Messenger RNA Vaccination Among Patients With a History of Kawasaki Disease

**DOI:** 10.1001/jamanetworkopen.2022.26236

**Published:** 2022-08-01

**Authors:** Mikayla Beckley, Aaron K. Olson, Michael A. Portman

**Affiliations:** Seattle Children’s Research Institute, Seattle, Washington; Seattle Children’s Research Institute, Seattle, Washington; Division of Cardiology, Department of Pediatrics, University of Washington, Seattle; Seattle Children’s Research Institute, Seattle, Washington; Division of Cardiology, Department of Pediatrics, University of Washington, Seattle

## Abstract

**IMPORTANCE:**

Kawasaki disease (KD) symptoms significantly overlap with multisystem inflammatory syndrome in children due to COVID-19. Patients with KD may be at risk for adverse outcomes from exposure to SARS-CoV-2 infection or vaccination.

**OBJECTIVE:**

To describe the outcomes of patients with KD to SARS-CoV-2 infection or vaccination.

**DESIGN, SETTING, AND PARTICIPANTS:**

This case series evaluated 2 cohorts using an existing KD database and reviewed individual electronic medical records for the period spanning January 1, 2020, through January 31, 2022, via electronic medical records that include Washington state immunization records. Vaccine cohort inclusion criteria consisted of being 21 years or younger at immunization and receiving 1 or more BNT162b2 (Pfizer-BioNTech) or messenger RNA (mRNA)–1273 (Moderna) vaccine doses. The COVID-19 cohort included patients 21 years or younger with positive polymerase chain reaction or nuclear capsid IgG findings for SARS-CoV-2. Participants included 826 patients from a preexisting KD database. One hundred fifty-three patients received at least 1 BNT162b2 or mRNA-1273 vaccine dose and were included in the mRNA vaccine cohort. Thirty-seven patients had positive test results for SARS-CoV-2 and were included in the COVID-19 cohort.

**EXPOSURES:**

SARS-CoV-2 vaccination and/or infection.

**MAIN OUTCOMES AND MEASURES:**

Adverse events after mRNA vaccination and/or COVID-19, including clinician visits, emergency department encounters, or hospitalizations.

**RESULTS:**

Among the 153 patients included in the mRNA vaccination cohort (mean [SD] age, 13.0 [4.3] years; 94 male [61.4%]), the BNT162b2 vaccine was provided for 143 (93.5%), and the remaining 10 (6.5%) received mRNA-1273 or a combination of both. Among patients in the vaccine cohort, 129 (84.3%) were fully vaccinated or received a third-dose booster. No clinically severe adverse events occurred, and there were no reports of vaccine-related hospitalizations or outpatient visits. The COVID-19 cohort included 37 patients (mean [SD] age, 11.0 [5.5] years; 22 male [59.5%]). No patients required hospitalization due to COVID-19. The most common symptoms included low-grade fever, fatigue, cough, and myalgia with resolution within a few days. Two patients, aged 9 and 19 years, had extended cough and fatigue for 3 to 4 weeks. One patient developed COVID-19 within 6 weeks of receiving intravenous immunoglobulin for KD.

**CONCLUSIONS AND RELEVANCE:**

These findings suggest that the mRNA vaccines may be safe and COVID-19 may not be severe for patients with a history of KD.

## Introduction

Kawasaki disease (KD) involves a unique autoinflammatory reaction in children and adolescents triggered by unknown pathogens or environmental agents. The inflammatory response is self-limited but can leave children with coronary artery wall abnormalities. Approximately 5% of patients with KD have a recurrence, also not linked to a specific environmental trigger.^1^ SARS-CoV-2 can elicit a profound immune response during the subacute infection phase in adults, which is associated with end-organ damage. Multisystem inflammatory syndrome in children also displays a late hyperinflammatory response during convalescence from COVID-19.^2^ The symptoms of multisystem inflammatory syndrome in children overlap with those of KD, and the delayed inflammatory response is poorly understood. Thus, concern exists that SARS-CoV-2 virus or messenger RNA (mRNA) vaccination could reactivate a hyperimmune response in patients with a history of KD. Patients with autoimmune syndromes have shown disease flares or reactivation after SARS-CoV-2 mRNA immunization.^3^ The pediatric vaccine trials have not evaluated safety and efficacy in specific populations with preceding or underlying diseases such as KD.^4,5^ Furthermore, limited information exists regarding COVID-19 severity in children with a history of KD, a potentially vulnerable population. We performed a case series observational study using an existing database to describe clinical outcomes of patients with KD after SARS-CoV-2 infection or vaccination.

## Methods

We assembled 2 cohorts using an existing KD database with approval from the institutional review board of the Seattle Children’s Research Institute. All 826 participants in the database fulfilled American Heart Association criteria for complete or incomplete KD diagnosis.^1^ We followed the Strengthening the Reporting of Observational Studies in Epidemiology (STROBE) reporting guidelines for clinical observational studies.

We extracted demographic data and reviewed individual electronic medical records for the period spanning January 1, 2020, through January 31, 2022, via the EpicCare Everywhere electronic medical record network (Epic Systems Corporation). In Washington State, 14 organizations participate in the network (eMethods in the Supplement). EpicCare Everywhere also receives data from the Washington State Immunization Service.

For the vaccine cohort, the inclusion criteria consisted of (1) being 21 years or younger at immunization and (2) receiving 1 or more documented BNT162b2 (Pfizer-BioNTech) or mRNA-1273 (Moderna) mRNA vaccine doses. The COVID-19 cohort included database enrollees 21 years or younger with a reported positive SARS-CoV-2 polymerase chain reaction or nuclear capsid IgG test result. We performed comprehensive medical record review using the EpicCare Everywhere network for potential reports of adverse events 30 days post vaccination and symptoms of COVID-19. Adverse events for the vaccine cohort were established by 2 pediatric cardiologists (A.K.O. and M.A.P.) and included (1) hospital encounters or clinic appointments, excluding well-child child visits; (2) messages from patients and/or parents to clinicians seeking medical advice; and (3) documentation of symptoms listed in the VAERS (Vaccine Adverse Event Reporting System) Table of Reportable Events Following Vaccination^6^ and those listed by both mRNA vaccine manufacturers within 30 days post immunization.^7^ We followed a similar process for those in the COVID-19 cohort, noting the presentation of symptoms prompting testing, duration of symptoms, and any hospitalizations related to COVID-19. We reviewed all potential adverse events in the context of each patient’s medical history to ensure that they were likely attributable to SARS-CoV-2 infection. Demographic statistics are reported as mean (SD) and median (95% CI).

## Results

**Figure 1.**
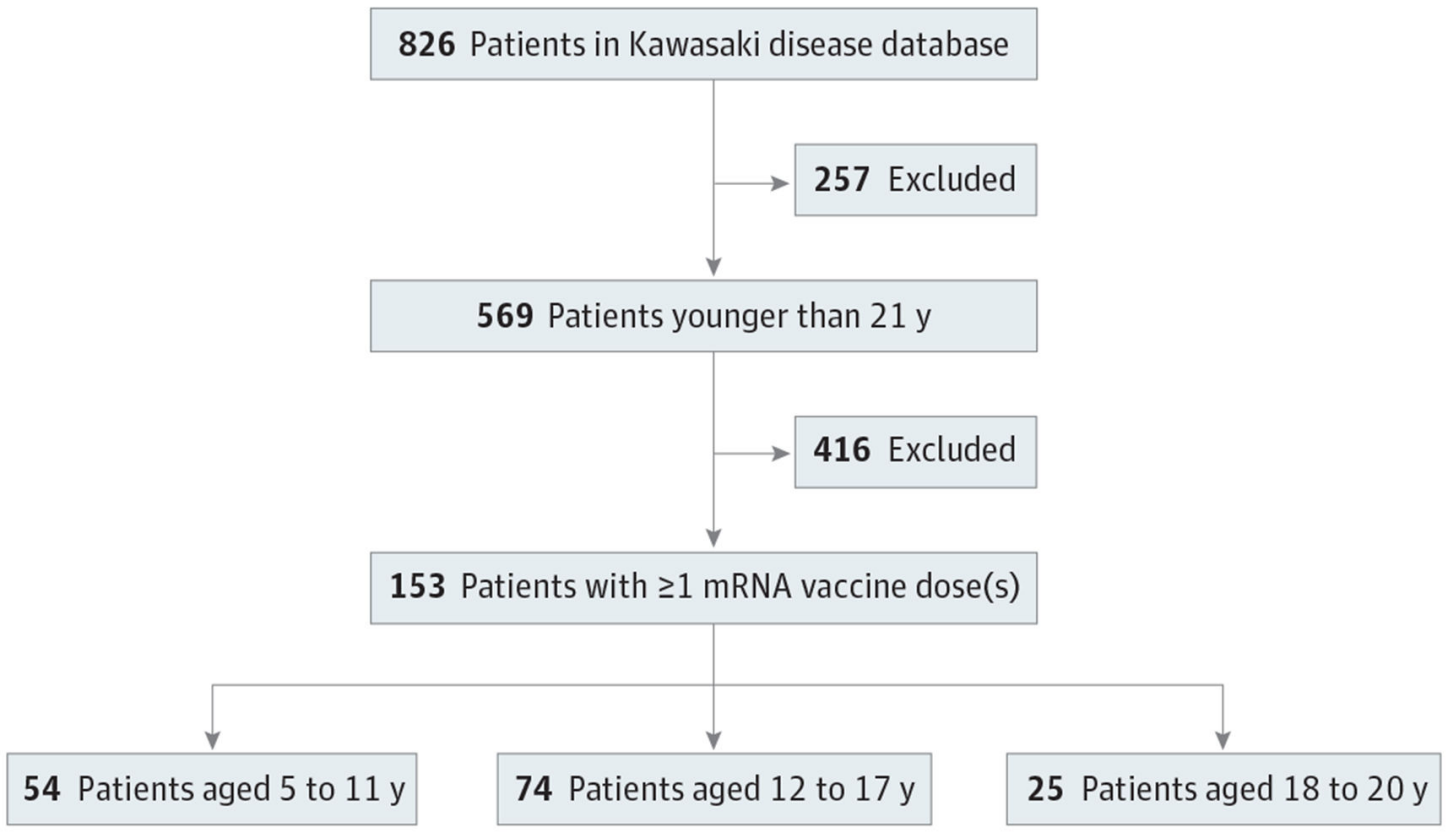
Flow Diagram of Study Identification Inclusion and Exclusion Criteria for Messenger RNA (mRNA) Vaccine Cohort

**Figure 2.**
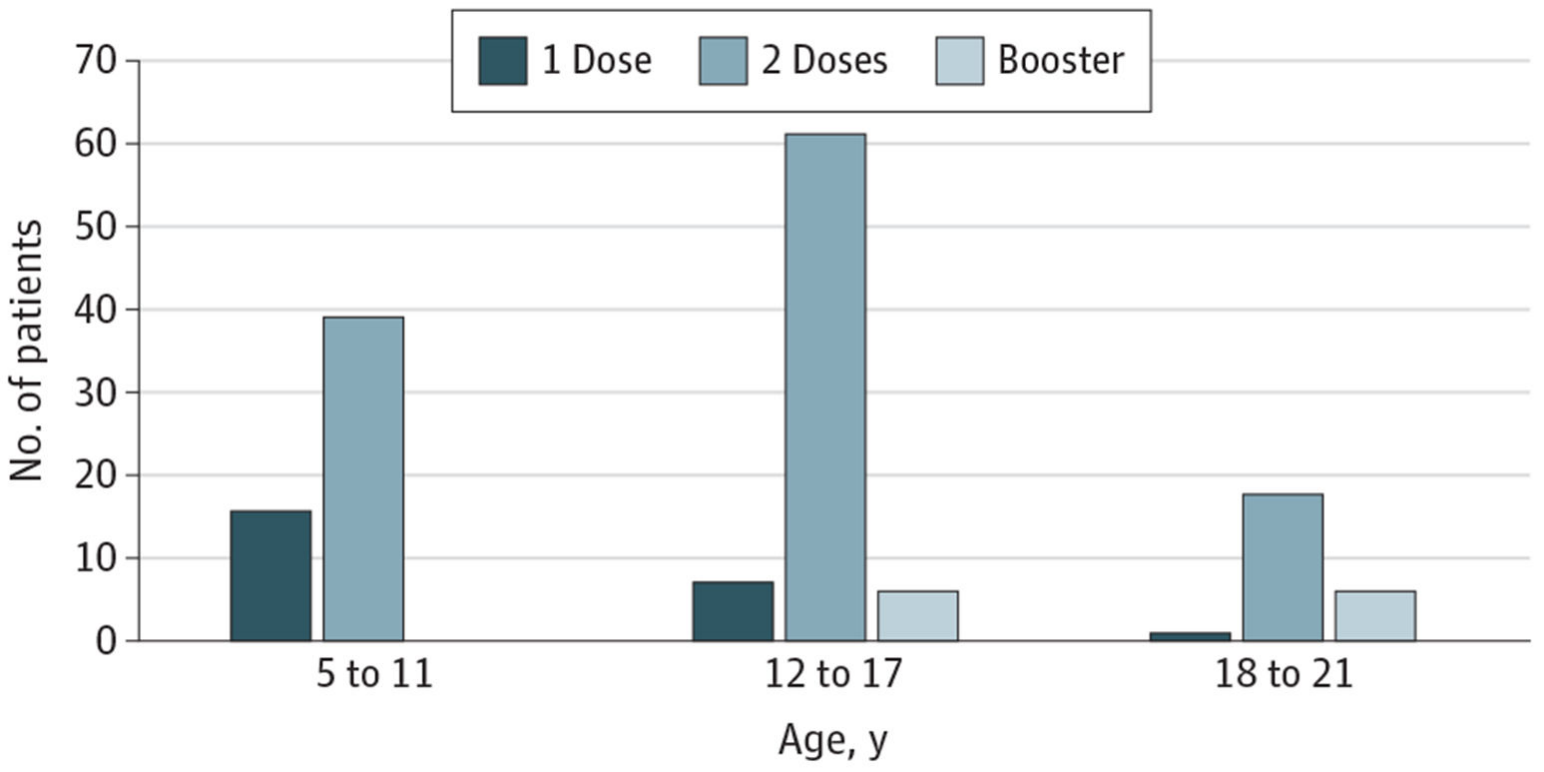
The Distribution of BNT162b2 and Messenger RNA-1273 Vaccine Doses Among Age Subgroups

The qualifying vaccine cohort contained 153 patients (mean [SD] age, 13.0 [4.3] years; 94 male [61.4%] and 59 female [38.6%]) (Figure 1), with age distribution and time from KD diagnosis shown in Table 1 and vaccine dose numbers by age in Figure 2. One hundred forty-three patients (93.5%) received the BNT162b2 vaccine, and the remaining 10 (6.5%) received mRNA-1273 or a combination. No clinically severe adverse events, clinician visits, emergency department encounters, or hospitalizations were reported within 30 days after immunization.

**Figure 3.**
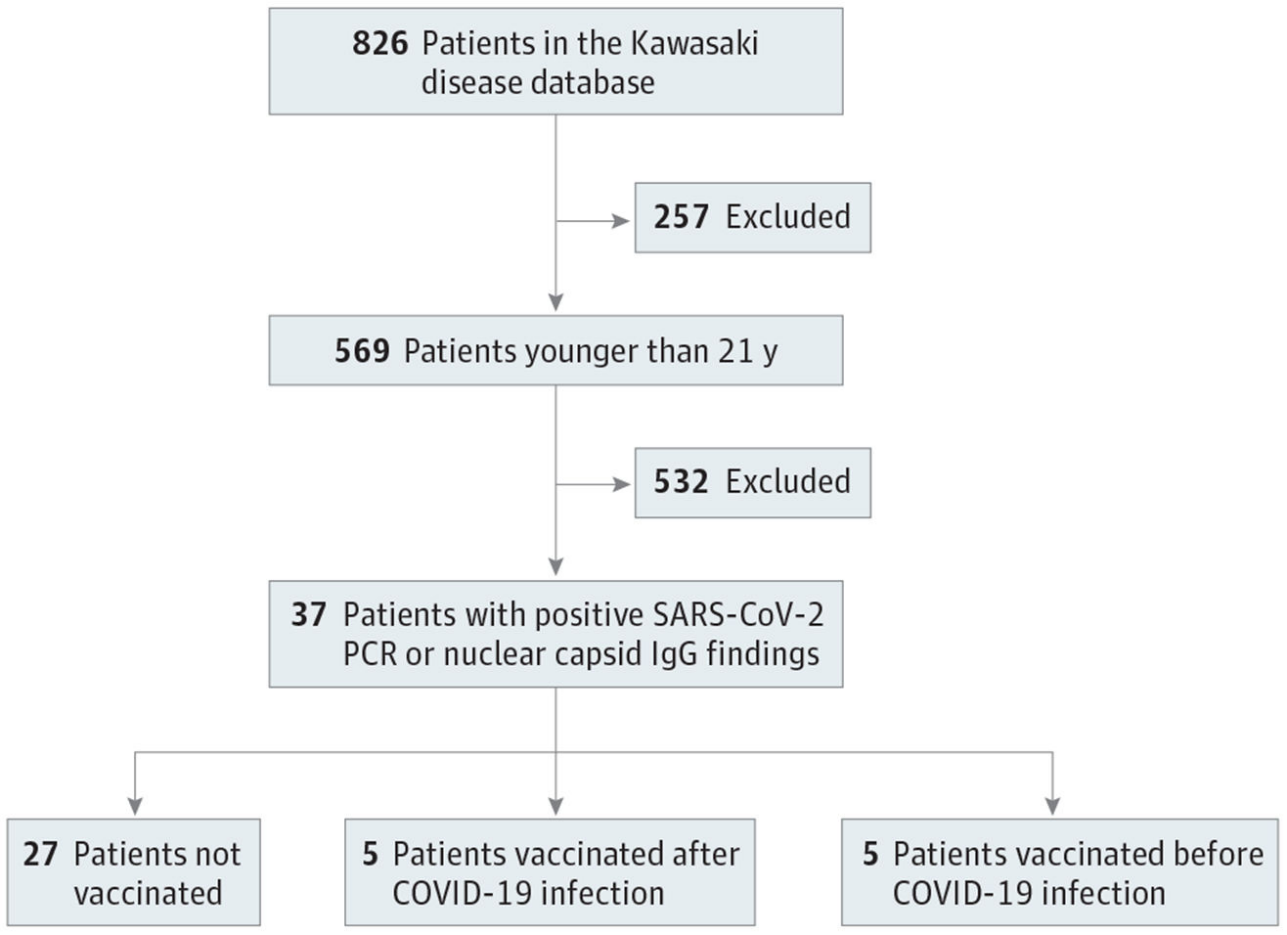
Flow Diagram of Study Identification Inclusion and Exclusion Criteria for SARS-CoV-2 Infection Cohort PCR indicates polymerase chain reaction.

Thirty-seven patients had positive test results for COVID-19 (mean [SD] age, 11.0 [5.5] years; 22 male [59.5%] and 15 female [40.5%]) (Figure 3), and 6 were treated for preceding KD after March 2020 with a negative SARS-CoV-2 polymerase chain reaction finding at that admission. No one required hospitalization. Among the 27 patients who were not vaccinated, 9 were asymptomatic after testing for exposure (Table 2). The most common symptoms included low grade fever, fatigue, cough, and myalgia with resolution within a few days. Two patients aged 9 and 19 years had extended cough and fatigue for 3 to 4 weeks. One patient developed COVID-19 within 6 weeks of receiving intravenous immunoglobulin for KD treatment. Nine patients had positive SARS-CoV-2 polymerase chain reaction findings between mid-December 2021 and mid-January 2022.

Ten patients with KD received at least 1 dose of the BNT162b2 vaccine and had positive test results for COVID-19. They all tolerated the mRNA vaccine administration and SARS-CoV-2 infection well, with no hospitalizations or adverse events noted in their respective electronic medical records. Five patients recovered from COVID-19 at least 3 months before receiving their first mRNA vaccine dose. Notably, 5 patients had a positive SARS-CoV-2 polymerase chain reaction findings despite previous vaccination. Four contracted COVID-19 4 to 7 months after receiving their second BNT162b2 dose. These patients all received intravenous immunoglobulin for KD treatment more than 5 years before their COVID-19 diagnosis.

## Discussion

This case series evaluates clinical outcomes of patients with KD after SARS-CoV-2 infection or vaccination. Few studies have evaluated COVID-19 vaccine safety in patients with autoinflammatory disease.^8,9^ Overall studies in adults suggest high toleration of COVID-19 mRNA vaccines. One survey-based study with nearly 3000 adult respondents^9^ reported that safety profile for mRNA vaccines resembled that for the general population. Only 5% of respondents reported requiring adjustment of disease-modifying medications associated with a flare.^9^ A Turkish single center study^10^ reported that children with rheumatological disease showed tolerance of the vaccines with few if any severe adverse events. Kawasaki disease is unique in that the clinical representation overlaps with SARS-COV-2-induced multisystem inflammatory syndrome in children, causing parental and clinician concern that exposure to SARS-CoV-2 infection or vaccination might reactivate the hyperinflammatory response considered a hallmark of KD. Although research is ongoing, both the pediatric syndromes also converge upon an interleukin 15– and interleukin 15 receptor α subunit–centric cytokine storm, suggestive of shared proximal pathways of immunopathogenesis.^11^

Our unique database, directed toward KD research, enabled this study’s performance. Data were compiled using the EpicCare Everywhere electronic medical record network. This network enables the exchange of medical record information between institutions participating in the network. This interoperability of patient health information allows for prompt retrieval of clinical data from outside organizations.^12^ By pulling electronic medical record data from other health care organizations, we performed robust analyses of immunizations and clinic encounters for each patient. Using these tools, we assembled relatively large cohorts for a rare disease with exposure to SARS-CoV-2 infection or vaccination.

Results of our study suggest that both COVID-19 and SARS-CoV-2 mRNA vaccination are well tolerated in patients with preceding KD. No severe complications occurred in either cohort with only 2 patients exhibiting more prolonged but relatively mild symptoms from COVID-19.

### Limitations

This study has limitations. Some data on patient mRNA vaccine status and COVID-19 symptoms may have not been reported or appropriately entered into individual electronic medical records. Although we had access to records at Seattle Children’s Hospital and those participating in EpicCare Everywhere, we were not able to capture vaccinations, hospital admissions, or infection status at other health care organizations or practices not participating in the network. However, in our region, any severe adverse events would likely be treated at an institution participating in EpicCare Everywhere.

## Conclusions

Findings of this case series suggest that SARS-CoV-2 infection and mRNA vaccination may be tolerated by patients with KD. Further research is needed to delineate the association between SARS-CoV-2 infection and vaccination and other autoimmune diseases.

## Supplementary Material

Supplement **eMethods.** Description of Data Sources

## Figures and Tables

**Table 1.** Demographic Characteristics of Vaccine Cohort by Age Subgroups

	Patient age subgroup		
Characteristic	5-11 y (n = 54)	12-17 y (n = 74)	18-21 y (n = 25)
Sex, No. (%)			
Male	32 (59.3)	48 (64.9)	14 (56.0)
Female	22 (40.7)	26 (35.1)	11 (44.0)
Age, at KD diagnosis, mo			
Mean (SD)	36.2 (23.2)	49.6 (35.9)	40.1 (26.4)
Median (95% CI)	31 (25-43)	37 (32-52)	35 (25-56)
Time between KD and first dose, mo			
Mean (SD)	65.6 (27.0)	122.9 (40.4)	188.1 (28.2)
Median (95% CI)	67 (57-77)	122 (117-138)	184 (170-204)

Abbreviation: KD, Kawasaki disease.

**Table 2.** Demographic Characteristics of COVID-19–Positive Cohort by mRNA Vaccine Status

	No. (%)		
Characteristic	Not vaccinated (n = 27)	Vaccinated before COVID-19 (n = 5)	Vaccinated after COVID-19 (n = 5)
Sex, No. (%)			
Male	15 (55.5)	3 (60.0)	4 (80.0)
Female	12 (44.4)	2 (40.0)	1 (20.0)
Age at KD diagnosis, mo			
Mean (SD)	41.9 (31.0)	40.8 (20.3)	67.6 (35.9)
Median (95% CI)	39 (25-50)	40 (10-62)	61 (32-127)
Time between KD and COVID-19, mo			
Mean (SD)	84.0 (61.3)	142.8 (20.3)	81.4 (33.6)
Median (95% CI)	77 (34-120)	131 (125-170)	71 (45-126)
Symptoms, No. (%)			
Mild	16 (59.3)	5 (100)	4 (80.0)
Moderatea^a^	2 (7.4)	0	0
Asymptomatic	9 (33.3)	0	1 (20.0)

Abbreviations: KD, Kawasaki disease; mRNA, messenger RNA.

a Persistent symptoms for 3 to 4 weeks, but no hospitalization.

## Key Points

### Question

What are the clinical outcomes of patients with a history of Kawasaki disease after exposure to SARS-CoV-2 infection or vaccination?

### Findings

In this case series of 153 patients with a history of Kawasaki disease, SARS-CoV-2 infection and/or vaccination was well tolerated, with no adverse events or hospital admissions documented.

### Meaning

These findings suggest that patients with history of Kawasaki disease tolerate SARS-CoV-2 infection or vaccination.
