# Supplementary material for: Tolerability of COVID-19 Infection and Messenger RNA Vaccination Among Patients With a History of Kawasaki Disease
Source: JAMA Netw Open. Author manuscript; Available in PMC 2022 Aug 31. (PMC9375169; doi:10.1001/jamanetworkopen.2022.26236)
Supplement: Supplement — eMethods. Description of Data Sources [file NIHMS1830296-supplement-Supplement.pdf]

Supplementary Online Content

Beckley M, Olson AK, Portman MA. Tolerability of COVID-19 infection and messenger RNA vaccination among patients with a history of Kawasaki disease. *JAMA Netw Open*. 2022;5(8):e2226236. doi:10.1001/jamanetworkopen.2022.26236

eMethods. Description of Data Sources
Reference

This supplementary material has been provided by the authors to give readers additional information about their work.

© 2022 Beckley M et al. *JAMA Network Open*.

eMethods. Description of Data Sources

Epic Care Everywhere[1]
The 14 participating health care organizations in the Epic Care Everywhere network across the state of Washington are listed below.

- Catholic Health Initiatives Pacific Region
- Confluence Health and Community Connect Partners
- Everett Clinic and Polyclinic
- Kaiser Permanente Washington
- MultiCare Health System
- Overlake Hospital Medical Center
- PeaceHealth
- Providence Health and Services Washington and Montana
- Seattle Children’s Hospital
- Skagit Regional Health
- The Vancouver Clinic
- UW Medicine-Washington
- Valley Medical Center
- Yakima Valley Farm Workers Clinic

© 2022 Beckley M et al. *JAMA Network Open*.

Reference

1. Care Everywhere. https://www.himss.org/resource-environmental-scan/care-everywhere

© 2022 Beckley M et al. *JAMA Network Open*.
